# Eyes as Windows: Unveiling Neuroinflammation in Multiple Sclerosis via Optic Neuritis and Uhthoff’s Phenomenon

**DOI:** 10.3390/diagnostics14192198

**Published:** 2024-10-02

**Authors:** Andreea Pleșa, Florina Anca Antochi, Mioara Laura Macovei, Alexandra-Georgiana Vîrlan, Ruxandra Georgescu, David-Ionuț Beuran, Săndica Nicoleta Bucurica, Carmen Adella Sîrbu, Any Axelerad, Florentina Cristina Pleșa

**Affiliations:** 1 Clinical Neurosciences Department, “Carol Davila” University of Medicine and Pharmacy, 050474 Bucharest, Romania; andreea.plesa@rez.umfcd.ro (A.P.);; 2 Neurology Department, “Dr. Carol Davila” Central Military Emergency University Hospital, 010825 Bucharest, Romania; ruxandrageorgescu03@gmail.com; 3 Neurology Department, University Emergency Hospital, 050098 Bucharest, Romania; 4 Ophthalmology Department, “Carol Davila” University of Medicine and Pharmacy, 050474 Bucharest, Romania; 5 Ophthalmology Department, “Dr. Carol Davila” Central Military Emergency University Hospital, 010825 Bucharest, Romania; davidbeuran96@gmail.com; 6 Pediatric Neurology Department, “Prof. Dr. Alexandru Obregia” Clinical Psychiatric Hospital, 041914 Bucharest, Romania; alexandra-georgiana.virlan@rez.umfcd.ro; 7 Departament 5, “Carol Davila” University of Medicine and Pharmacy, 050474 Bucharest, Romania; sandica.bucurica@umfcd.ro; 8 Academy of Romanian Scientists, 050045 Bucharest, Romania; 9 Department of Neurology, General Medicine Faculty, Ovidius University, 900470 Constanta, Romania; 10 Department of Neurology, St. Andrew County Clinical Emergency Hospital of Constanta, 900591 Constanta, Romania

**Keywords:** multiple sclerosis, optic neuritis, Uhthoff’s phenomenon, Lhermitte’s sign, neuroinflammation, neurodegeneration, demyelination, visual acuity impairment, dyschromatopsia, ophthalmological examination, early diagnosis

## Abstract

**Background/Objectives**: This study investigated the frequency and timing of optic neuritis (ON) episodes in relation to the onset of multiple sclerosis (MS) and examined the occurrence of Uhthoff’s phenomenon and Lhermitte’s sign to understand their roles in early diagnosis and disease progression. **Methods**: A longitudinal study was conducted with 127 MS patients. Clinical data, including ophthalmological examinations for ON, were collected and questionnaires assessed the presence of Uhthoff’s phenomenon and Lhermitte’s sign. **Results:** Results showed that 37% of patients experienced demyelinating retrobulbar ON, with 25.53% having ON as the initial symptom of MS. Residual visual acuity impairment (below 20/40) and dyschromatopsia were reported by 25.53% and 17.02% of patients, respectively. Uhthoff’s phenomenon and Lhermitte’s sign were present in 26.77% and 36.22% of patients, respectively. The findings underscore the importance of early ophthalmological assessments in diagnosing MS, as ON can be an initial indicator of the disease. **Conclusions:** The study highlights the need for precise diagnostic tools and personalized therapeutic strategies focused on specific biomarkers and pathways involved in neuroinflammation and demyelination. Early diagnosis through vigilant ophthalmologic evaluation can lead to interventions that significantly alter disease progression, improving patient outcomes and quality of life.

## 1. Introduction

Multiple sclerosis (MS) is a chronic inflammatory and degenerative disease of the central nervous system, with a progressive evolution that can lead to significant disability. This disease mainly affects young adults, being the main cause of non-traumatic disability among them [1]. Worldwide, 2.8 million people are diagnosed with multiple sclerosis and 30,000 of them are under the age of 18 [2].

Patients with multiple sclerosis have an extensive spectrum of neurological signs and symptoms as a result of variable localization of the central nervous system lesions. The most common of these symptoms are: vision loss, double vision, visual field disorders, nystagmus, speech and swallowing disorders, sensory impairments, gait disorders, ataxia, spasticity, and bladder and sphincter disorders [3].

Ophthalmic symptoms appear frequently, sometimes even before the neurological signs. About 50% of all patients diagnosed with multiple sclerosis will develop one or more episodes of acute demyelinating optic neuritis [4]. Even the first case of multiple sclerosis ever described in the literature was one of a young Dutch woman who suffered from visual impairment as part of the natural course of the disease [5]. The most common manifestations are due to optic neuritis, nystagmus, and internuclear ophthalmoplegia [6,7].

The causes of optic neuritis (ON) are varied and can be classified into different categories based on their underlying mechanisms. These include demyelinating, infectious, inflammatory, and other less common etiologies (detailed in Table 1 [8]). Understanding the full spectrum of potential causes is essential for accurate diagnosis and management.

In patients with demyelinating ON, it is crucial to consider the differential diagnosis of multiple sclerosis (MS), neuromyelitis optica spectrum disorder (NMOSD), and the newly discovered myelin oligodendrocyte glycoprotein antibody-associated disease (MOGAD). All of these conditions can present with similar initial symptoms, such as acute visual impairment due to optic nerve inflammation. However, they differ significantly in their underlying pathophysiology, clinical manifestations, prognosis, and treatment strategies [9]. The characteristics and distinctions of optic neuritis in MS, NMOSD, and MOGAD are detailed in Table 2 [10].

Although profound vision loss is unusual in multiple sclerosis, even a subtle visual deterioration may be hazardous in patients who already have sensory or motor dysfunctions.

Identifying the most common ophthalmological manifestations that may occur, together with a careful clinical and paraclinical examination of a young patient who complains of visual disturbances can lead to an early diagnosis of multiple sclerosis.

The Uhthoff phenomenon and Lhermitte’s sign are clinical features observed in multiple sclerosis. Recognizing these signs can serve as a clinical argument for early neurological assessment, facilitating timely intervention.

The Uhthoff phenomenon, first described by ophthalmologist Wilhelm Uhthoff in 1890, is most commonly noticed in MS, with 60% to 80% of MS patients experiencing this phenomenon when exposed to heat. However, it can also occur in other optic disorders affecting the afferent pathways [11]. Uhthoff’s phenomenon is characterized by a stereotyped worsening of neurological symptoms, especially visual disturbances, that is temporary and lasts less than 24 h [12]. It is triggered by elevated body temperature due to exercise, fever, or hot weather. It is important to differentiate this transient worsening from an exacerbation of MS or a true relapse.

Uhthoff’s phenomenon is most commonly associated with multiple sclerosis (MS); however, other rare conditions may present with similar symptoms. Although these conditions are uncommon, they should be included in the differential diagnosis to ensure a comprehensive evaluation and appropriate management. A detailed list of conditions to consider in the differential diagnosis of Uhthoff’s phenomenon is provided in Table 3 [12].

Lhermitte’s sign, characterized by an electric shock-like sensation that radiates down the spine and into the limbs when the neck is flexed, is a common manifestation in patients with MS, appearing in up to 41% of cases [13]. However, it is not specific to MS and can be found in other pathologies involving the cervical spinal cord. Table 4 categorizes the differential diagnosis of Lhermitte’s sign, emphasizing the importance of considering these diverse conditions in clinical practice. Research indicates that its prevalence among MS patients is notably lower compared to those with neuromyelitis optica (NMO) [14]. This sensation results from miscommunication between demyelinated nerves in the dorsal column of the spinal cord, particularly at the cervical level [15].

The aim of this study was to determine the frequency of ON episodes and their timing relative to the onset of MS, as well as to assess the frequency of Uhthoff phenomenon and Lhermitte’s sign in these patients.

## 2. Materials and Methods

This was a descriptive, observational, retrospective, and longitudinal study involving 127 patients diagnosed with multiple sclerosis (MS) who were undergoing treatment with disease-modifying therapy. The patients were admitted to the Neurology Department at “Dr. Carol Davila” Central Military Emergency University Hospital between July 2022 and June 2024.

Patient selection followed specific inclusion and exclusion criteria. Patients were excluded if they had experienced an acute relapse of optic neuritis (ON) within the last six months, as this could affect the assessment of residual impairment. Additionally, patients with a disease duration shorter than one year were excluded to ensure that the study encompassed chronic cases, including primary progressive MS. Finally, patients with other eye conditions, such as glaucoma, which could independently cause visual impairment, were also excluded from the study.

Data collection was conducted retrospectively using patient records from the hospital’s archives. The clinical data had been previously gathered through anamnesis, clinical examination, and paraclinical investigations. An eye examination, which included visual acuity testing with a 4 m Snellen chart and color vision testing using Ishihara Color Blindness Test Plates, was performed by an ophthalmologist. Additionally, the presence of Uhthoff phenomenon and Lhermitte’s sign was assessed using a self-administered questionnaire completed by the patients during their medical visits.

The collected data were stored and subsequently processed and statistically analyzed using Microsoft Office Excel 2021. Quantitative variables were analyzed using descriptive statistics and measures of central tendency (mean), with standard errors and deviations calculated. The results of the categorical variable analysis were graphically represented using pie charts and bar charts. Quantitative variables were converted into categorical variables and also graphically represented in the same way.

To determine the statistical significance of the processed data, the predictive value (*p*) was calculated using the Student’s *t*-test for quantitative variables and the Chi-square test for qualitative variables. A *p*-value ≤ 0.05 was considered statistically significant.

## 3. Results

### 3.1. Clinical and Demographic Characteristics

Our analysis of the clinical data revealed a significant prevalence of MS-related ON, with 37% (47 out of 127) of patients having a history of this condition. We divided the patients into two categories: those with and those without a history of ON, and evaluated their age, gender, residency, age at onset of the disease, type of MS, and treatment.

Patients with ON were younger at the onset of MS (31.60 ± 9.38 years) compared to those without (35.76 ± 10.51 years), the difference being statistically significant (*p* = 0.039). This finding aligns with previous studies suggesting that ON often presents earlier in the disease course of MS. There were no significant differences between the two groups concerning other characteristics such as gender, residency, type of MS, and treatment (Table 5).

When analyzing the demographic data for patients with a history of ON, we found out that the mean age was 41.87 ± 11.958 years (range 22 to 68 years). Seventy-four percent of patients were women and the mean age at the onset of MS was 31.60 ± 9.380 years. These data were consistent with the literature, considering that MS predominantly affects young women [16].

Most of the patients lived in urban areas, with an urban-to-rural ratio of 4:1. The higher proportion of urban residents in our sample (4:1 urban-to-rural ratio) can be attributed to the greater ease with which urban residents can access hospitals and doctors compared to rural residents. This is noteworthy, especially considering that the urban and rural populations in Romania are almost equal.

The majority of patients (96%) presented with the relapsing–remitting form of MS, while 4% had the secondary progressive form. This distribution aligns with expectations, given that relapsing–remitting MS is the most common form of the disease [16]. Regarding treatment, beta interferon was the most prescribed drug, followed by ocrelizumab and teriflunomide.

To assess disease severity, we used the Expanded Disability Status Scale (EDSS), which ranges from 0 to 10 in 0.5 increments based on neurological examination and patient history [17,18]. The mean EDSS score in the ON group was 2.20 ± 1.51, slightly lower than the score in the non-ON group (2.35 ± 1.80). Despite the higher score in the latter group, patients with ON showed a considerable impact on overall disability, indicating the significant role of visual symptoms in the disease burden.

### 3.2. Optic Neuritis Characteristics

**Figure 1:**
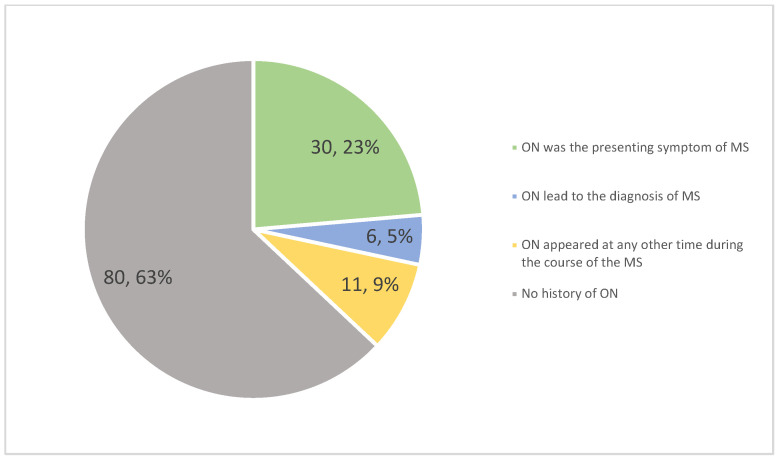
Distribution of optic neuritis (ON) in multiple sclerosis (MS) patients.

Visual impairment was reported as the initial symptom of MS by 63% of patients with ON (30 out of 47). This represented 25.53% of all study participants, underscoring the importance of comprehensive ophthalmological examinations for early diagnosis. In an additional six cases, ON led to the diagnosis of MS, even though it was not the first relapse (see Figure 1).

**Figure 2:**
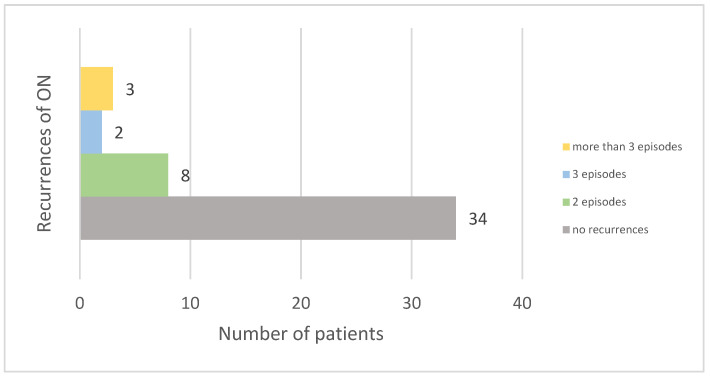
Frequency of optic neuritis recurrences among patients.

The majority of patients (72.34%) did not experience recurrences of ON. Among those who did, eight had two episodes, two had three episodes, and three had more than three episodes. Four patients experienced bilateral optic nerve involvement at disease onset (see Figure 2).

**Figure 3:**
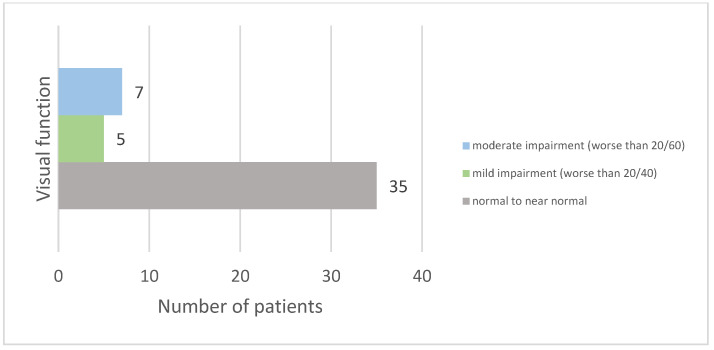
Distribution of visual acuity outcomes after optic neuritis.

Following ON, 74.47% of patients experienced recovery with normal or near-normal visual acuity. Furthermore, 25.53% had lasting visual impairment, with their best-corrected visual acuity in the worst-affected eye being less than 20/40. Visual dysfunction was classified according to the *International Classification of Diseases 11* (2018), with five patients (10.63%) having mild impairment and seven (14.89%) having moderate impairment (see Figure 3).

**Figure 4:**
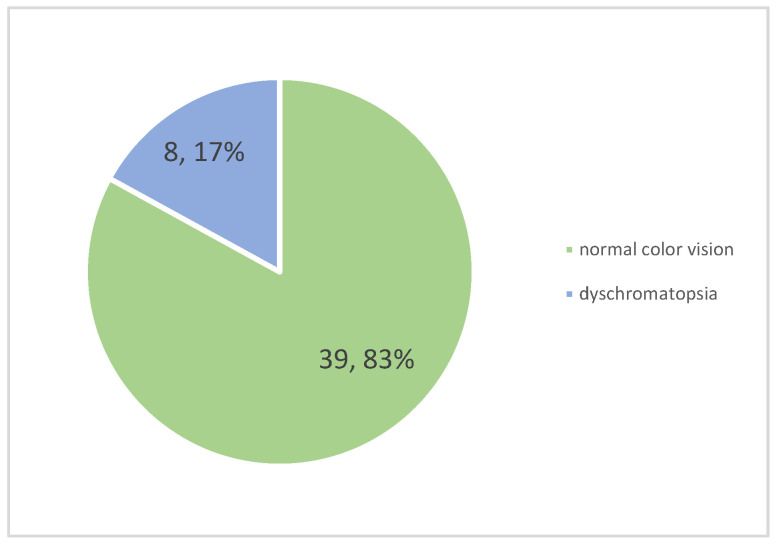
Frequency of dyschromatopsia among patients.

Color vision dysfunction, assessed via Ishihara plates, was found in eight patients (17.02%) and is represented in Figure 4. Bilateral dyschromatopsia was present in two patients, while the remaining cases involved unilateral impairment. The most common type was red–green color vision deficiency, present in seven patients.

### 3.3. Uhthoff Phenomenon and Lhermitte’s Sign

**Figure 5:**
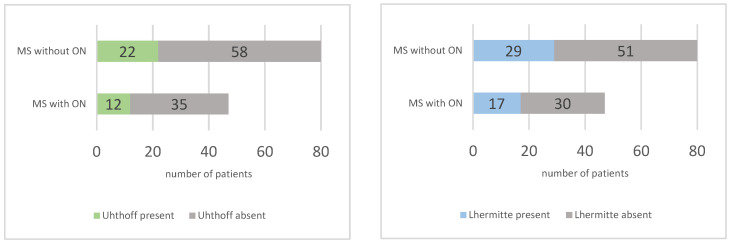
Prevalence of Uhthoff phenomenon (**left**) and Lhermitte sign (**right**) in multiple sclerosis (MS) patients with and without optic neuritis (ON).

The presence of the Uhthoff phenomenon and Lhermitte’s sign was evaluated via a self-administered questionnaire. The Uhthoff phenomenon was observed in 34 out of 127 patients (26.77%), with a slightly higher frequency in the group without ON (27.5% vs. 25.53%, RR = 0.93). Lhermitte’s sign was present in 46 out of 127 patients (36.22%), with no significant difference between the two groups (36.17% in the group with ON and 36.25% in the group without, RR = 0.99). The prevalence of these phenomena is represented in Figure 5. These symptoms are common in MS and further underscore the complex and varied symptomatology of the disease.

## 4. Discussion

### 4.1. Optic Neuritis in Multiple Sclerosis

Optic neuritis (ON) in multiple sclerosis arises from complex molecular mechanisms involving immune-mediated damage to the optic nerve. The optic nerve is part of the central nervous system (CNS). It consists of approximately 1.2 million axons originating from the retinal ganglion cells [19]. These ganglion cell axons are unmyelinated fibers along the optic nerve head, becoming myelinated immediately posterior to the lamina cribrosa [20]. Oligodendrocytes are responsible for myelination and the maintenance of saltatory conduction, which facilitates efficient nerve impulse transmission along the axons within the CNS. The localization of oligodendrocytes in the posterior compartment explains the pattern of inflammatory damage to the optic nerve during ON, as retinal inflammation is not typical. In MS, retrobulbar ON is characteristic [21].

In MS, damage to the optic nerve occurs through several mechanisms, including demyelination mediated by the death of oligodendroglial cells, activation of glial cells (such as microglia and astrocytes), and axonal degeneration [21]. The body’s natural response to demyelination involves the activation of immunomodulatory networks that limit inflammation and initiate repair processes, resulting in partial remyelination and clinical remission [22]. However, endogenous remyelination has limitations. Remyelinated axons often have thinner myelin sheaths and shorter internodal lengths, leading to slower axonal conduction velocities. Moreover, recurrent demyelination can further impair remyelination processes, resulting in permanently demyelinated axons and chronic damage in MS [23].

ON is a common clinical manifestation in patients with MS. Various studies have reported differing prevalence rates of ON in MS, reflecting variability due to study design, follow-up period, and population characteristics. Studies indicate that optic neuritis (ON) occurs in approximately half of multiple sclerosis (MS) patients over the course of the disease [24,25]. In our study, 37% of patients experienced ON. This lower prevalence compared to the literature may be influenced by the variability in follow-up duration, as our cohort included patients diagnosed between one and over twenty years ago. While some patients were followed for a long period, others, particularly those more recently diagnosed, had shorter follow-up times. This design limits the ability to capture the lifetime prevalence of ON for all patients. Given the known frequency of ON in MS, it is possible that additional cases may emerge with further follow-up. Additionally, ON may be underrecognized, particularly in milder or transient cases where medical attention may not have been sought. Given the high prevalence, the presence of ophthalmological manifestations in a young patient requires a complete ophthalmological examination and referral to a neurologist for early treatment and diagnosis of a possible MS onset.

ON is often the first symptom of MS. Studies have reported that ON is the initial presenting symptom in up to 25% of cases [26]. Our results are consistent with this, as we found that 25.53% of MS patients had ON as their initial symptom. However, older studies reported lower rates, such as 15–20% [27,28]. This discrepancy could be due to the under-recognition of isolated ON, leading to delayed referrals to neurologists. Additionally, symptoms of ON are often transient, and patients might not seek immediate medical attention, delaying diagnosis until subsequent relapses occur. Moreover, variability might arise from differences in healthcare access, diagnostic criteria, and awareness of ON among primary care providers. The high rate of visual impairment as an initial symptom of MS underscores the need for early and thorough ophthalmological evaluation and referral to a neurologist in suspected cases.

Recurrent ON is not uncommon. In the ten-year follow-up of the Optic Neuritis Treatment Trial, recurrences of ON were observed in 35% of the patients [29]. In our study, 27.66% of patients with a history of ON experienced recurrences. The lower rate in our study can also be explained by the shorter follow-up period for some of the patients.

There is a risk of residual visual impairment following an episode of ON. In the ten-year follow-up of the Optic Neuritis Treatment Trial, 9% of patients had a visual acuity worse than 20/40 in the affected eye [29]. It is important to note that in this study, not all patients with ON developed MS. Visual function was generally poorer in patients with multiple sclerosis. Since the reported percentage refers to the entire group of patients, it may underestimate the rate of residual visual dysfunction specifically in MS patients. This could explain the much higher rate of residual visual dysfunction observed in our group (25.53%). Additionally, some patients were followed for a longer period and experienced recurrences of ON, which likely contributed to the higher rate of residual visual impairment. A limitation of the study in assessing residual visual dysfunction is that we only used the Snellen chart for assessment. We did not evaluate optic nerve atrophy using OCT or MRI measurements, which can reveal structural damage that correlates with visual dysfunction.

In a small study, color vision was evaluated in a group of patients with ON, and color blindness, as diagnosed by the Ishihara test, was found in four out of forty-four patients (20.45%) [30]. In our study, color vision dysfunction was identified in a similar percentage, 17.02%. This suggests that color vision deficits are a common, though often overlooked, consequence of ON in MS.

The frequency of ON recurrences and the residual impairment observed in our patients highlight the disease burden in this population. Continuous ophthalmological monitoring is crucial for patients with MS, given that ON can occur at any stage of the disease.

The management of multiple sclerosis (MS) involves a comprehensive approach tailored to the individual’s disease form, severity, and other factors such as age, overall health, and response to previous treatments and family planning considerations. Disease-modifying therapies (DMTs) are at the forefront of MS management, with proven efficacy in reducing the frequency and severity of relapses, slowing disease progression, and limiting the development of new lesions as seen via MRI. The early initiation of disease-modifying therapies can slow the progression of the disease and reduce the degree of disability in these patients [31]. The treatment of acute relapse in multiple sclerosis typically involves corticosteroid therapy to reduce inflammation, significantly shorten the duration of the relapse, and improve symptoms. Prompt administration is crucial. For severe relapses that do not respond to steroid treatment, plasmapheresis can be an effective alternative. This is of utmost importance when addressing ON, as the resulting visual dysfunction can be a highly debilitating condition. Prompt and effective treatment is essential to reduce the impact on vision and overall quality of life.

Symptomatic therapy plays a crucial role in improving the quality of life for individuals with MS, addressing issues like spasticity, pain, fatigue, and bladder dysfunction [32]. Although these treatments do not modify the disease course, they are essential for maintaining daily comfort and functioning. Interest is also growing in complementary and alternative therapies, such as cannabis, vitamin D, dietary changes, physical activity, exercise, and yoga [33,34,35]. While preliminary evidence suggests potential benefits, more research is needed to confirm their effectiveness and safety. Neurorehabilitation is a cornerstone of MS management, aiming to optimize physical and cognitive function through a comprehensive, multidisciplinary approach [36,37].

### 4.2. Uhthoff’s Phenomenon and Lhermitte Sign

Uhthoff’s Phenomenon occurs because demyelination causes the nodes of Ranvier, which are rich in sodium channels, to widen [38]. This widening disrupts the organization of sodium channels and impairs depolarization. As a compensatory mechanism, new sodium channels form along the axonal membrane, but they have altered properties [39]. Even a minimal increase in temperature (as little as 0.5 °C) is enough to close these sodium channels and halt depolarization. Furthermore, the exposed potassium channels lead to hyperpolarization. These combined effects result in delayed or blocked conduction that clinically manifest as worsening MS symptoms [12].

The underlying mechanism behind Lhermitte’s sign involves a disruption in normal nerve conduction due to myelin loss, which is crucial for the rapid transmission of nerve impulses. In demyelinated fibers, conduction becomes erratic and can lead to the misfiring of sensory signals. The pathophysiology also includes activation of neuropathic pain pathways, influenced by the impaired function of inhibitory GABAergic interneurons, which normally regulate sensory input and prevent excessive excitability [40]. Additionally, microglia, the central nervous system’s immune cells, play a key role. Their activation increases the production of inflammatory cytokines, which exacerbate the inflammatory environment in MS, exacerbating the abnormal neuronal signaling and leading to the clinical manifestation of Lhermitte’s sign [13].

Uhthoff’s phenomenon is reported to affect approximately 60% to 80% of MS patients in various studies [12]. Lhermitte’s sign is observed in 9 to 41% of MS patients [13]. In our study, Uhthoff’s phenomenon was observed in 26.77% of patients, while Lhermitte’s sign was present in 36.22%. These findings are consistent with the prevalence range for Lhermitte’s sign but notably lower for Uhthoff’s phenomenon compared to the literature. This discrepancy might be attributed to our use of a standardized self-assessment questionnaire, which could result in underreporting due to patients’ difficulty recognizing or recalling their symptoms.

## 5. Conclusions

MS, a neurological disorder which mainly affects women and young people, frequently causes ocular manifestations, sometimes as the first clinical sign of the disease. Uhthoff phenomenon and Lhermitte’s sign are clinical phenomena that may occur in MS and can lead to timely diagnosis.

The intricate molecular mechanisms underlying MS significantly contribute to neuroinflammation and the development of conditions such as ON, Uhthoff’s phenomenon, and Lhermitte’s sign. Understanding these molecular mechanisms is crucial for advancing diagnostic and monitoring techniques in MS. By identifying specific biomarkers and pathways involved in neuroinflammation and demyelination, clinicians can develop more accurate diagnostic tools and personalized therapeutic strategies. Tailored treatments that target these molecular processes could potentially enhance remyelination, reduce neuroinflammation, and prevent the progression of chronic damage, ultimately improving the quality of life for individuals with multiple sclerosis.

Our study adds to existing knowledge by providing detailed clinical and demographic insights into Romanian MS patients with ON, emphasizing the importance of early diagnosis and comprehensive management of visual symptoms. The role of ophthalmologists in the early detection and referral to neurologists is critical, as it ensures timely and effective treatment for MS patients. Early diagnosis facilitated by vigilant ophthalmological evaluation can lead to interventions that may significantly alter the disease’s progression and improve patient outcomes and quality of life.

## Figures and Tables

**Table 1:** Causes of optic neuritis.

Demyelinating	Infections	Inflammatory	Others
Multiple sclerosis NMOSD MOGAD	Viral Bacterial	Parainfectious Sarcoidosis CRION Systemic autoimmune diseases	Compressive Genetic causes Toxic and metabolic Trauma

Abbreviations: NMOSD: Neuromyelitis optica spectrum disorder; MOGAD: myelin oligodendrocyte glycoprotein antibody-associated disease; CRION: chronic relapsing inflammatory optic neuropathy.

**Table 2:** Comparison of ON Features in MS, NMOSD, and MOGAD.

ON Features	MS	NMOSD	MOGAD
Bilateral presentation	rare	common	common
Visual acuity impairment	mild to moderate	moderate to severe	moderate to severe
Ocular pain	common	rare	common
Optic nerve involvement	segmental (<50% of optic nerve)	extensive (>50% of optic nerve)	extensive (>50% of optic nerve)
Optic chiasm involvement	infrequent	common	uncommon
	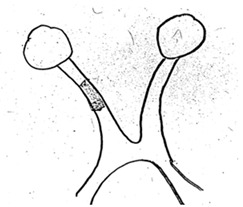	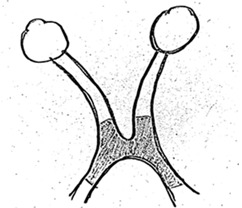	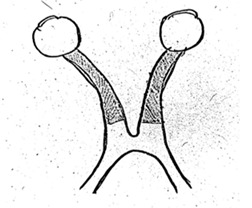
Perineuritis	absent or mild	less common	common
RNFL measured via OCT	typically normal in acute phase	typically normal in acute phase	acutely thickening
Relapse recovery	intermediate	generally poor	usually good

Abbreviations: ON: optic nerve; MS: multiple sclerosis; NMOSD: neuromyelitis optica spectrum disorder; MOGAD: myelin oligodendrocyte glycoprotein antibody disease; RNFL: retinal nerve fiber layer; OCT: optical coherence tomography.

**Table 3:** Differential diagnosis of Uhthoff’s phenomenon.

Category	Specific Causes
Demyelinating Diseases	MS, NMOSD
Autoimmune Disorders	Antiphospholipid antibody syndrome, Behçet’s disease, CNS lupus, CNS vasculitis, Sjogren’s syndrome
Infectious Diseases	HIV, HTLV, Lyme disease
Hematologic Conditions	CNS lymphoma
Nutritional Deficiencies	Copper deficiency
Genetic Disorders	Leukodystrophies
Vascular Disorders	Small vessel disease
Other Conditions	Sarcoidosis, osmotic demyelination syndrome

Abbreviations: MS: multiple sclerosis; NMOSD: neuromyelitis optica spectrum disorder; CNS: central nervous system; HIV: human immunodeficiency virus; HTLV: human T-lymphotropic virus.

**Table 4:** Differential diagnosis of Lhermitte’s sign.

Category	Specific Causes
Demyelinating Diseases	MS
Autoimmune Disorders	Transverse myelitis, CNS lupus, Behçet’s disease
Infectious Diseases	Herpes zoster toxicity, parasitic invasion of the cord
Nutritional Deficiencies	Vitamin B12 deficiency
Toxic/Drug-related	Radiation myelopathy, high dose chemoradiation (cisplatin), nitric oxide toxicity
Mechanical/Structural	Tumor, radiculopathy, cervical spondylitis, Arnold–Chiari malformation, syringomyelia, trauma, arachnoiditis, post-dural puncture headache

Abbreviations: MS: multiple sclerosis; CNS: central nervous system.

**Table 5:** General characteristics of the cohort.

Parameter		Group 1—Patients with ON *N* = 47	Group 2—Patients without ON *N* = 80	*p* Value
Age	Mean age ± SD	41.87 ± 11.958 years	44.44 ± 10.918 years	0.223 (NS)
Gender N, %	Male	12 (26%)	25 (31%)	0.557 (NS)
	Female	35 (74%)	55 (69%)	
Residency	Urban	37 (79%)	69 (86%)	0.273 (NS)
	Rural	10 (21%)	11 (14%)	
MS age of onset	Mean age of onset ± SD	31.60 ± 9.380 years	35.76 ± 10.511 years	0.039 (S)
Type of MS	Recurrent remissive	45 (96%)	65 (81%)	0.132 (NS)
	Primary progressive	0 (0%)	8 (10%)	
	Secondary progressive	2 (4%)	6 (9%)	
EDSS score	Mean EDSS ± SD	2.20 ± 1.51	2.35 ± 1.801	0.639 (NS)
Treatment	Beta-interferon	18 (38%)	18 (23%)	-
	Ocrelizumab	7 (15%)	18 (23%)	
	Teriflunomide	5 (11%)	13 (16%)	
	Natalizumab	5 (11%)	6 (8%)	
	Glatiramer acetate	4 (9%)	3 (4%)	
	Cladribine	4 (9%)	6 (8%)	
	Dimethyl fumarate	2 (4%)	7 (9%)	
	Fingolimod	1 (2%)	5 (6%)	
	Siponimod	1 (2%)	4 (5%)	

Abbreviations: ON: optic nerve; MS: multiple sclerosis; EDSS: Expanded Disability Status Scale; SD: standard deviation. NS: not significant; S: significant.

## Data Availability

The data presented in this study are available on request from the corresponding author due to privacy reasons.
